# Examining Differences in Health-Related Technology Use between Millennial and Older Generations of Caregivers

**DOI:** 10.3390/nursrep14040192

**Published:** 2024-09-24

**Authors:** Virginia T. Gallagher, Shannon E. Reilly, David Martin, Carol Manning, Kelly M. Shaffer

**Affiliations:** 1 Department of Neurology, School of Medicine, University of Virginia, P.O. Box 801018, Charlottesville, VA 22908, USA; sr6hd@virginia.edu (S.E.R.);; 2 Claude Moore Health Sciences Library, University of Virginia Health System, Charlottesville, VA 22908, USA; dnm5ca@virginia.edu; 3 Center for Behavioral Health and Technology, School of Medicine, University of Virginia, Charlottesville, VA 22908, USA

**Keywords:** caregiving, smartphone, technology, digital health, Millennial, generation

## Abstract

Background/Objective: Caregivers from the Millennial generation are an emerging and understudied group of unpaid care providers in America who may benefit from digitally delivered support. To inform the design/tailoring of interventions for this group, we aimed to understand how Millennials may differ from other generations of caregivers regarding digital health-related technology use. Methods: Using the Health Information National Trends Survey (HINTS), Version 6, we conducted a cross-sectional study comparing health technology access and use across four generations of unpaid caregivers (n = 545; Millennials, Gen X, Baby Boomers, and Silent Generation) of adults with chronic conditions using chi-square and Kruskal–Wallis non-parametric tests. Results: Compared to Baby Boomer and Silent Generation caregivers, Millennial caregivers more frequently reported having a cellular internet connection, using a wearable activity device, a health/wellness mobile application, choosing telehealth appointments for convenience, and most frequently used social media in general and to view health-related videos (ps < 0.005). Additionally, Millennials were more likely to report possessing a smartphone (compared to Gen X and Silent Generation) and more frequently used social media for peer interaction about health (compared to all older generations; ps < 0.005). Conclusion: Millennials differ from older generations of caregivers regarding health-related technology access and use, which may have implications for intervention design and tailoring.

## 1. Introduction

There are approximately 47.9 million unpaid caregivers in the United States supporting adult family members or friends with health or functional needs [1]. These caregivers are at risk for adverse health and quality of life outcomes due to the physical and emotional demands of caregiving [2,3,4].

**Figure 1:**
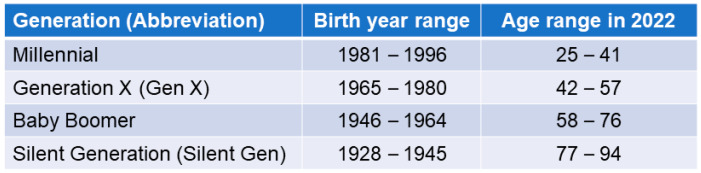
Generation definitions.

Millennial caregivers are an emerging cohort of over 10 million individuals in the United States alone. This group is understudied and has unique traits compared to other generations of caregivers (Figure 1) [1].

In the general population, Millennials have higher rates of psychological distress and select chronic health problems relative to older generations [5,6]. For Millennial caregivers specifically, the time demands and burden of caregiving may be greater relative to older generations. Millennial caregivers are more likely than other age bands of caregivers to be simultaneously working and providing care [7,8,9,10,11]. Millennial caregivers may also be called upon to provide care for more family members than previous generations because decreased fertility rates over the last 50 years have yielded fewer siblings to share the care burden for supporting parents and extended relatives [8,10]. In sum, Millennial caregivers are a group at particular risk of adverse impacts from caregiving. Establishing efficacious interventions and supports for this emerging cohort of caregivers is essential.

Given the differences across generations of caregivers, it is problematic that previous caregiving interventions have largely been tested among Gen X or Baby Boomer generations [12]. Nonetheless, existing studies have demonstrated that interventions can effectively address caregivers’ burden, depression, stress, knowledge, and self-efficacy among older generations [13,14]. The widespread use of these interventions has been limited [15]. Delivering these interventions via the Internet is expected to improve reach and uptake, particularly among younger generations of caregivers [16]. Understanding how Millennial caregivers’ technology use compares with that of older generations will help inform how digital health interventions may need to be modified for the greatest acceptability and feasibility for this rising caregiver cohort.

Based on research in the general population, we expect that Millennial caregivers will be more likely than older generations to embrace digital health technology and interventions. The current literature suggests that younger generations (e.g., Millennials) are more likely than older generations (e.g., Baby Boomers) to use social media, podcasts, and blogs for healthcare purposes [17], to prioritize telehealth medical care versus in-person care [18], and to readily adopt app-based digital activity trackers [19]. While we expect generational differences in health technology use in the general population to extend to caregiver populations, prior research has demonstrated that caregivers have different health technology use compared to non-caregivers. For example, across ages, caregivers are 1.3× more likely to use the internet to search health-related topics compared to non-caregivers [20]. This underlines the importance of investigating health technology use in different age groups among caregivers specifically.

Little literature is available regarding caregivers’ use of health-related technology by age group or generation. A single study revealed that older caregivers are less likely to use health technology compared to younger caregivers [21]. However, no studies to date have specifically investigated generational differences in caregivers’ health-related technology use. Generational status offers an important, novel heuristic for tailoring interventions towards a large group of individuals with many shared characteristics, life-cycle stages, attitudes, behaviors, and technology use [22,23].

To establish necessary evidence for appropriate intervention design and tailoring for the rising generation of caregivers, the goal of this study was to evaluate the extent to which Millennial caregivers of adults with chronic conditions differ from other generations of caregivers in health-related technology access and use. Specifically, we compared Millennial caregivers to older generations of caregivers on internet access methods, digital health engagement, and telehealth use through a large national U.S. survey of diverse adults. We anticipate that the results will inform considerations of accessibility of internet-delivered caregiver support interventions.

## 2. Materials and Methods

### 2.1. Study Design and Participants

This is a cross-sectional analysis that uses data from the Health Information National Trends Survey, Version 6, Cycle 1 (HINTS 6). HINTS is a nationally representative survey administered by the National Cancer Institute to adults aged 18 or older living in the United States who speak English or Spanish. HINTS 6 was conducted from 7 March to 8 November 2022 [24]. HINTS focuses on understanding health information support needs in the population. In addition to standard HINTS content, HINTS 6 included a special interest assessment focused on the use of telehealth. HINTS 6 data are publicly available from https://hints.cancer.gov/data/download-data.aspx (accessed on 1 July 2023). The authors did not contribute to the collection of HINTS 6 data. The HINTS 6 survey was designated “exempt research” under 45-CFR-46.104 and approved by the Westat IRB on 5 October 2021 (Project: 6632.03.51). HINTS 6 also received a “Not Human Subjects Research” determination from the NIH Office of IRB Operations on 11/162021 (iRIS: 562715).

This analysis exclusively reports data from the 545 participants who self-identified as the primary, unpaid caregiver for an adult with a chronic health condition. This was operationalized as participants answering affirmatively to the item, “Are you currently caring for or making health care decisions for someone with a medical, behavioral, disability or some other condition?” Caregivers who exclusively reported providing care for a child were excluded from this analysis due to the distinct nature of providing care for a child with special care needs [25,26]. Participants were also excluded if their age (and therefore generational status) was missing.

### 2.2. Measures

#### 2.2.1. Demographic, Socioeconomic, and Caregiving Characteristics

Participants self-reported demographic information including age, sex, sexual orientation, race/ethnicity, educational background, occupational status, marital status, household residents (total number of adults and presence of children), and household income attitudes. Race/ethnicity, degree of rurality, and household income attitude were selected as key socioeconomic variables that may impact digital health access and engagement based on prior studies [27,28]. Participants’ degree of rurality was measured by the USDA 2010 Primary Rural-Urban Community Area Code. Attitude regarding household income was selected over monetary household income amounts, as unadjusted monetary income may be misleading due to extreme variance in the cost of living in various parts of the United States [29]. Select categories of these key socioeconomic variables were collapsed for analysis, to aim for n > 5 per cell by generation.

#### 2.2.2. Age Categorization into Generations

Generation was defined by post hoc categorization of participants’ self-reported age in 2022 into the following categories [22]: Silent Generation (age ≥ 77 at the time of data collection, i.e., born 1928–1945; n = 46), Baby Boomers (age 58–76, born 1946–1964; n = 277), Gen X (age 42–57, born 1965–1980; n = 149), and Millennials (age ≤ 41, born 1981–1996; n = 66). Seven participants aged 18–25, who are considered part of Generation Z, were included with Millennials (total n = 73). See Figure 1.

#### 2.2.3. Dependent Variables

Outcomes of interest were selected from the HINTS 6 based on their relevance to informing digital health intervention development and dissemination for caregivers. Variable selection was also informed by prior work examining health technology use among caregivers [21]. Specifically, internet access mechanisms, smartphone possession, social media use, health/wellness app and devise use, and telehealth use were deemed relevant to inform intervention modality, platform, feasibility, and acceptability of future caregiver interventions. Outcomes were assessed by specified single-item questions detailed in Table 1, also available online [24]. Response options in Table 1 denote the categorization of responses used in analyses. Some response options were collapsed across categories to ensure adequate cell size (n > 5) for analysis and to aid interpretation of results.

***General Internet Access.*** Internet use, cellular network internet access, smartphone possession, and broad social media use variables were selected to inform caregivers’ access to and use of the internet and social media more broadly. Cellular network internet access was selected based on its ability to inform the appropriateness of intervention delivery via smartphones that require internet access out of the home or workplace.

***Digital Health Engagement*.** Digital health engagement variables were selected to evaluate the extent to which Millennials and other generations of caregivers may already be using health-related mobile applications, activity trackers, and social media.

***Telehealth Use*.** Telehealth engagement variables were selected to understand Millennials’ and other caregiving generations’ use of more formal telehealth services, such as communicating with healthcare providers or attending virtual appointments.

### 2.3. Statistical Analyses

Analyses were conducted using SPSS version 28. Descriptive statistics for demographics, lifestyle factors, and caregiving characteristics included means with standard deviations, medians with interquartile range, or frequencies, as appropriate. Generational differences in key socioeconomic factors (race/ethnicity, rurality, and household income attitude) were examined via chi-square tests of independence to inform sensitivity analyses. Chi-square tests of independence tested whether caregivers’ generation was related to categorical outcomes and Kruskal–Wallis non-parametric tests were used for ordinal outcomes due to the non-normal distribution of data. Given the relationship between access/use of technology and environmental social determinants of health [27,28], post hoc sensitivity regression analyses were conducted to determine whether significant generational differences in outcomes of interest could be explained by other key socioeconomic factors (i.e., race/ethnicity, degree of rurality, and household income attitudes [see Table 2]). Specific sensitivity regression analyses were either ordinal (for ordinal outcomes), binary logistic (for dichotomous outcomes), or multinomial logistic (for categorical outcomes with 2+ categories).

An alpha threshold of *p* < 0.05 was used for main hypothesis testing. Where group-wise differences were detected, post hoc pairwise comparisons were made between Millennial caregivers versus the other generational groups. post hoc pairwise differences were interpreted using a Bonferroni correction of *p* < 0.016 for multiple (three) comparisons. Taylor’s Series variance estimation was used to estimate standard errors, which reduces bias and, therefore, type I error. These procedures are in accordance with published HINTS analysis recommendations [24].

## 3. Results

Sample generational demographics and lifestyle factors are presented in Table 2. Caregiving characteristics are presented in Table 3. Analysis of generational differences in key socioeconomic variables (see Table 2) revealed generational differences in race/ethnicity proportions; pairwise comparisons revealed the proportion of Non-Hispanic White caregivers was smaller in the Millennial generation relative to the Baby Boomer and Silent Generations (ps < 0.001). There was also a difference across generations in attitudes about household income; pairwise comparisons revealed Millennials were more frequently finding it difficult to get by on their income compared to Baby Boomers (*p* = 0.003). No generational differences in rurality were detected. Across outcome variables, responses were not ascertained (missing) for 0.00–4.40% of cases (average of 1.53% missing values per variable).

### 3.1. General Internet Access

**Figure 2:**
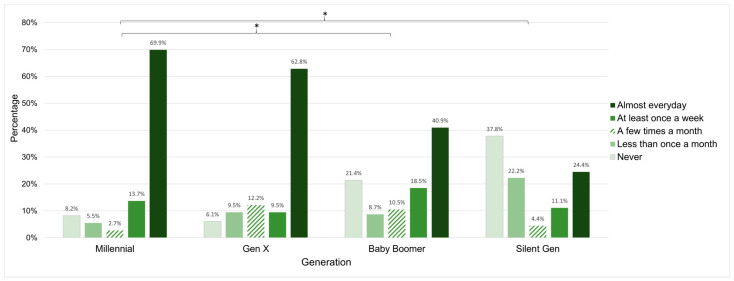
Social media: Millennial caregivers report more frequent social media use versus Baby Boomer and Silent Generation caregivers (* ps < 0.016).

See Table 4 for General Internet Access results. Overall, Millennial caregivers more frequently reported accessing the Internet compared to the Silent Generation only. Of participants who reported accessing the Internet (n = 470, 86.24% of the total sample), Millennial caregivers more frequently reported accessing the Internet through a cellular network compared to Baby Boomers and the Silent Generation. Among caregivers who access the internet, Millennials more frequently reported having a smartphone compared to Gen X and the Silent Generation. Finally, most Millennials caregivers reported visiting a social media site almost every day in the last year, which was more frequent than Baby Boomers and the Silent Generation (see Figure 2). 

### 3.2. Digital Health Engagement

See Table 5 for Digital Health Engagement results. Overall, Millennials more frequently reported using a health/wellness app and a wearable device for health/activity tracking compared to both Baby Boomers and the Silent Generation. Relative to these two generations, Millennials also more frequently used social media for viewing health-related videos. Relative to all other generations, Millennials more frequently used social media to interact with others with similar health or medical issues.

### 3.3. Telehealth Engagement

See Table 6 for Telehealth Engagement results. Millennial caregivers more frequently reported using the Internet to make an appointment with a healthcare provider compared to Baby Boomer and Silent Generation caregivers. Among caregivers who chose telehealth appointments for their own healthcare needs, Millennials more frequently cited convenience as a reason they chose telehealth compared to Baby Boomer and Silent generation caregivers.

### 3.4. Sensitivity Analyses

Sensitivity analyses using ordinal (for ordinal outcomes), binary logistic (for dichotomous outcomes), and multinomial logistic (for categorical outcomes with 2+ categories) regression indicated that race/ethnicity, rurality, and household income attitude do not account for generational differences in general internet access, digital health engagement, or telehealth engagement variables.

## 4. Discussion

This analysis of a large, national U.S. survey demonstrates how Millennials differ from other generations of caregivers in their health-related technology use. Particularly when compared to Silent Generation and Baby Boomer caregivers, Millennial caregivers more frequently reported internet use, cellular internet access, smartphone possession, social media use, health/wellness app use, wearable health/activity trackers, and using telehealth healthcare options for convenience. While there were many significant differences between Millennials compared to both Baby Boomers or Silent Generation, there were fewer differences between Millennials and Gen X, in keeping with broader population trends [23]. Nonetheless, relative to Gen X, Millennials were more likely to possess a smartphone and more frequently interacted with peers on social media about health-related topics. Collectively, the findings from this study have important implications for the design, tailoring, and testing of digital interventions for younger caregivers.

Existing digital interventions for caregivers have primarily been piloted with caregivers from Gen X and Baby Boomer generations [12]. A leading suspected cause of dropout from digital caregiver intervention trials is low digital literacy, which is more common among older generations [12]. Given that in this study, Millennials were more likely than Gen X or Baby Boomers to use digital devices, apps, and social media platforms, it is possible that Millennial caregivers would remain more engaged with digital caregiver interventions than documented in prior studies with older generations.

Supportive interventions that are delivered over the Internet via smartphone may be particularly accessible and acceptable for Millennial caregivers. Specifically, over 95% of the surveyed Millennial caregivers reported accessing the Internet via a cellular network, more than any older generation (54–91%). Further, 100% of Millennial caregiver responders reported possessing a smartphone, relative to 82–93% of other caregiver generations. Findings are consistent with a 2023 Pew Research survey that revealed that American adults aged 30–49 have higher rates of smartphone ownership (97%), relative to older age bands (89% ownership for ages 50–64; 76% ownership for ages 65+) [30]. Additionally, Millennial caregivers in this study were 1.3×–1.5× more likely to have used a health/wellness mobile application in the previous year compared to Silent Generation and Baby Boomer caregivers. Further, two-thirds of Millennial caregivers in this study reported that convenience was a reason they chose telehealth over in-person medical care. These collective findings indicate that interventions delivered via smartphone can meet Millennial caregivers where they already are and capitalize on existing positive associations of telehealth use for health-related concerns in this time-scarce cohort.

While there is often concern that delivering interventions via smartphone application may perpetuate inequities in access to care among lower-resourced individuals [31], our results suggest that among Millennial caregivers, smartphone possession was high even among those experiencing subjective financial distress. Specifically, nearly 33% of Millennial caregivers in this study reported difficulty getting by on their income (Table 2), yet, as previously stated, 95% reported cellular internet access (Table 3). In contrast, 16% of Baby Boomers reported difficulty getting by on their current income, but just 77% reported cellular internet access. Findings are consistent with national longitudinal survey data revealing that younger Americans are now very likely to have a smartphone and mobile internet access regardless of income [30]. While national data indicate that smartphone possession and internet access are still less frequently reported among Black/African Americans (relative to non-Hispanic White Americans) and among rural Americans (relative to suburban/urban Americans), race/ethnicity, rurality, and income attitude did not explain generational differences in health technology use in this study. Therefore, interventions delivered by a smartphone may be well-suited to serve this younger cohort of caregivers, despite the fact that they are more financially unstable than other generations [11].

This study also demonstrates the potential of social media platforms for recruiting and engaging Millennial caregivers in research studies. Nearly 70% of Millennial caregivers in this study reported visiting a social media site such as Facebook, Twitter (now X), TikTok, YouTube, or Instagram almost daily over the last year, and an additional 13% reported at least weekly use. Instagram may be a particularly fruitful recruitment tool as approximately 50% of nearly 2.4 billion Instagram users are Millennials [32]. Social media recruitment strategies have the potential to engage clinical research participants who otherwise do not have access to research studies being conducted at academic medical centers [33].

Furthermore, given Millennial caregivers’ frequent social media use, social media may be a worthy platform to consider for intervention delivery in this cohort, particularly when considering a social support component. Indeed, emerging research has revealed that family caregivers who use social media for peer social support often gain a sense of social inclusion and belonging and that they appreciate that online platforms offer anonymity, 24 h access, and flexibility [34]. Our results suggest that Millennial caregivers already more frequently interact with people with similar health or medical issues using social media or online forums compared to all older generations, suggesting that this cohort may be more ready and willing to share private information about their caregiving experience or their health online compared to previous generations. To date, however, there is no published research on whether the use and impact of social media support groups vary based on a caregiver’s age or generation [35]. Given the gender and racial/ethnic diversity of Millennial caregivers, future research must examine how useful and effective social media support mechanisms will be for this diverse subgroup of caregivers.

Beyond social support components of caregiver interventions, social media may be a promising platform for delivering psychoeducational content for caregiving needs. In particular, we found that over half of Millennials viewed health-related videos on social media at least once per month. As such, perhaps videos and infographics delivered via social media or other online platforms may be feasible and effective ways to package psychoeducational content for Millennial caregivers in light of the decreased attention span for print content in this generation relative to previous generations [36,37].

Findings from this study must be interpreted in light of the broad definition of caregiver for inclusion (i.e., caring for or making healthcare decisions for an adult with a medical, behavioral, disability, or some other condition). Using this definition, it is possible that a caregiver may be making healthcare decisions for someone but may have low or nonexistent day-to-day engagement in assisting that individual with activities of daily living. Future studies aiming to characterize technology-related factors among caregivers should include a more specific definition of caregiver and more thorough assessments of caregiving responsibilities, including frequency, intensity, and duration of caregiving. Such factors may be important because care recipients who cannot be safely left alone may have caregivers who are more dependent on digitally delivered health services. Finally, the HINTS 6 survey did not include specific questions regarding the frequency of health/wellness app use, types of health/wellness apps used, or specific social media platforms used, and all data are self-reported.

## 5. Conclusions

Millennial caregivers are a growing cohort who face increased time demands relative to prior caregiver generations. Digital interventions may be particularly useful to support this emerging generation of caregivers. To that end, the results revealed that compared to select older generations, Millennials more frequently reported cellular internet access, smartphone possession, use of wearable activity devices and health/wellness mobile applications, social media use, and choosing telehealth medical care options because of convenience. These findings suggest that digital interventions or intervention components delivered via smartphone, smartphone mobile applications, and/or social media may be particularly useful among Millennial caregivers. Nursing clinicians who encounter caregivers of adults with chronic conditions may consider using Internet-based supportive tools, particularly among younger generations of caregivers.

## Figures and Tables

**Table 1:** Health-related technology dependent variables.

	Variable (Original HINTS 6 Question Number)	Survey Question	(Re-)Coded Response Options *
**General Internet Access**	Internet use (B1)	Do you ever go online to access the Internet or World Wide Web, or to send and receive e-mail?	*Yes*
			*No*
	Cellular internet access (B2c)	When you use the Internet, do you access it through a cellular network (i.e., phone, 3G/4G/5G)?	*Yes*
			*No*
	Smartphone possession (B6)	Please indicate if you have [the] following	*Smartphone*
			*No smartphone*
	Social media use (B12a)	Sometimes people use the Internet to connect with other people online through social media. Examples of social media sites include Facebook, Twitter, TikTok, YouTube, and Instagram. In the past 12 months, how often did you… Visit a social media site?	*Never (0)*
			*Less than once a month (1)*
			*A few times a month (2)*
			*At least once a week (3)*
			*Almost everyday (4)*
**Digital Health Engagement**	Health/wellness app use (B7)	[For participants who had a smart device] In the past 12 months, have you used a health or wellness app on your table or smartphone?	*Yes*
			*No (No or I do not have any health apps on my tablet or smart phone)*
	Wearable health device use (B8)	In the past 12 months, have you used an electronic wearable device to monitor or track your health or activity? For example a Fitbit, Apple Watch, or Garmin Vivofit	*Yes*
			*No*
	Social media peer health interaction (B12d)	In the past 12 months how often did you… Interact with people who have similar health or medical issues on social media or online forums	*Never (0)*
			*Less than once a month (1)*
			*A few times a month (2)*
			*At least once a week (3)*
			*Almost everyday (4)*
	Social media health-related videos (B12e)	In the past 12 months how often did you… Watch a health-related video on a social media site (for example, YouTube).	*Never (0)*
			*Less than once a month (1)*
			*A few times a month (2)*
			*At least once a week (3)*
			*Almost everyday (4)*
**Telehealth Use**	Telehealth use types (B3)	In the past 12 months, have you used the Internet to take care of any of the following health-related needs?	*Look for health or medical information*
			*Send a message to a healthcare provider*
			*View medical test results*
			*Make an appointment with a healthcare provider*
	Telehealth visits (D1)	In the past 12 months, did you receive care from a doctor or health professional using telehealth	*Yes (Video and/or phone)*
			*No*
	Telehealth non-use reason (D3)	[For participants who were offered telehealth but declined] Did you chose not to participate in a telehealth visit for any of the following reasons?	*I preferred to have the appointment(s) in person*
			*I was concerned about the privacy of telehealth visits*
			*I thought the telehealth technology would be difficult to use*
	Telehealth use reason (D4)	[For participants who used telehealth] Why did you choose a telehealth visit(s) for yourself?	*Provider recommended or required… use [of] telehealth*
			*I wanted advice about whether I needed in-person medical care*
			*I wanted to avoid possible infection at the docotor’s office*
			*It was more convenient than going to the doctor*
			*I could include family or other caregivers in my appointment*

Note. * Some response options were re-coded from original dataset to facilitate interpretability of results.

**Table 2:** Sample demographics.

		Millennials	Gen X	Baby Boomers	Silent Gen
		n = 73	n = 149	n = 277	n = 46
**Age, Mean (SD)**		32.96 (6.10)	51.44 (4.22)	66.53 (5.44)	82.87 (4.80)
**Female sex ^a^, n (%)**		47 (69.12%)	111 (75.51%)	182 (66.91%)	21 (45.65%)
**Sexual orientation**					
	Heterosexual, n (%)	49 (74.24%)	135 (93.75%)	256 (96.24%)	38 (95.00%)
	Gay, Lesbian, Bisexual, or Other, n (%)	17 (23.29%)	9 (6.40%)	10 (3.61%)	2 (5.00%)
**Race/ethnicity** [overall X^2^ (9) = 45.865, *p* < 0.001]					
	Non-Hispanic White, n (%)	22 (32.84%)	63 (47.37%)	158 (62.20%)	33 (64.71%)
	Non-Hispanic Black or African American, n (%)	13 (20.97%)	28 (21.05%)	33 (12.99%)	1 (1.96%)
	Hispanic, n (%)	10 (16.63%)	23 (17.29%)	33 (12.99%)	5 (9.80%)
	Non-Hispanic Asian, n (%) ^b^	9 (14.52%)	7 (5.26%)	11 (4.33%)	1 (1.96%)
	Non-Hispanic Other, n (%) ^b^	7 (9.59%)	7 (4.70%)	6 (2.16%)	2 (4.35%)
**Education**					
	High school or less, n (%)	12 (17.65%)	25 (16.89%)	48 (17.65%)	12 (26.09%)
	More than high school, n (%)	56 (82.35%)	123 (83.11%)	224 (82.35%)	34 (73.91%)
**Occupational Status**					
	Usually work ≥35 h/week, n (%) Yes	48 (70.59%)	99 (66.89%)	96 (35.29%)	3 (6.67%)
**Marital status**					
	Married or living as married, n (%)	32 (48.48%)	98 (66.67%)	182 (66.91%)	37 (80.43%)
	Single/separated/divorced/widowed, n (%)	34 (51.52%)	49 (33.33%)	90 (33.09%)	9 (19.57%)
**Household residents**					
	Number of adults (including self), Mean (SD)	2.26 (1.12)	2.38 (1.09)	2.21 (1.06)	2.04 (0.67)
	Children under 18 in the household, n (%)	40 (58.82%)	45 (30.82%)	12 (4.44%)	1 (2.33%)
**Rurality** [overall X^2^ (6) = 7.594, *p* = 0.055]					
	Metropolitan, n (%)	65 (89.04%)	131 (87.92%)	226 (81.59%)	34 (73.91%)
	Micropolitan, n (%) ^c^	4 (5.48%)	9 (6.04%)	28 (10.11%)	9 (19.57%)
	Small town, n (%)^c^	4 (5.48%)	5 (3.36%)	10 (3.61%)	2 (4.35%)
	Rural, n (%)^c^	0 (0.00%)	4 (2.68%)	13 (4.69%)	1 (2.17%)
**Attitude about household income** [overall X^2^ (6) = 16.133, *p* = 0.013]					
	Living comfortably, n (%)	23 (34.85%)	57 (39.04%)	128 (47.76%)	20 (46.51%)
	Getting by, n (%)	19 (28.79%)	49 (33.56%)	94 (35.07%)	17 (39.53%)
	Finding it difficult to get by, n (%) ^d^	15 (20.55%)	29 (19.46%)	35 (12.64%)	6 (13.04%)
	Finding it very difficulty to get by, n (%) ^d^	9 (12.33%)	11 (7.38%)	11 (3.97%)	0 (0.00%)

Comparisons across generations are reported above for key socioeconomic variables used in sensitivity analyses. ^a^ refers to sex assigned at birth; ^b^ categories were collapsed into “Non-Hispanic Other” for analyses; ^c^ collapsed into “Micropolitan or smaller” for analyses; ^d^ collapsed into “Finding it difficult or very difficult to get by” for analyses; n may not add up to total group size due to missing data.

**Table 3:** Caregiving characteristics.

**Caregivers’ relationship with care-recipient(s) ^a^**					
	Parent(s) only, n (%)	25 (34.25%)	70 (46.98%)	107 (38.63%)	1 (2.17%)
	Spouse/partner only, n (%)	13 (17.81%)	17 (11.41%)	81 (29.24%)	28 (60.87%)
	Another family member only, n (%)	10 (13.70%)	27 (18.12%)	43 (15.52%)	9 (19.57%)
	Friend/other non-relative only, n (%)	2 (2.74%)	3 (2.01%)	15 (5.42%)	3 (6.52%)
**Multiple care-recipient relationships**					
	Yes, n (%)	23 (31.51%)	32 (21.48%)	31 (11.19%)	5 (10.87%)
**Primary care-recipient condition(s) ^b^**					
	Cancer only, n (%)	3 (4.11%)	6 (4.03%)	11 (3.97%)	1 (2.17%)
	Alzheimer’s, confusion, dementia, forgetfulness, brain injury, stroke, or other neurological issue only, n (%)	5 (6.85%)	20 (13.42%)	38 (17.33%)	8 (17.39%)
	A long-term illness (i.e., high blood pressure, hypertension, diabetes, heart disease, heart attack, lung disease, or emphysema) only, n (%)	9 (12.33%)	23 (15.44%)	26 (9.39%)	7 (15.22%)
	Difficulty moving around such as an orthopedic issue, a musculoskeletal issue, or an aging-related issue only, n (%)	5 (6.85%)	9 (6.04%)	28 (10.11%)	3 (6.52%)
	Mental health issue, substance abuse, intellectual/developmental abnormality, n (%)	7 (9.59%)	15 (10.07%)	12 (4.33%)	2 (4.35%)
	Multiple conditions, n (%)	43 (58.90%)	73 (48.99%)	143 (51.62%)	25 (54.35%)

Comparisons across generations are reported above for key socioeconomic variables used in sensitivity analyses. ^a^ Caregivers were asked if they provided care or made healthcare decisions for any of the following care recipients (check all that apply). ^b^ Caregivers were asked to specify the condition(s) of the person for whom they provide the most care; when more than one condition was marked, responses were re-coded into “Multiple conditions”; n may not add up to total group size due to missing data.

**Table 4:** General internet access by caregiver generation.

	Millennials	Gen X	Baby Boomers	Silent Gen		
	n = 73	n = 149	n = 277	n = 46	X^2^ (df) ^a^	*p*
Internet use, n (%) Yes	65 (89.04%)	136 (91.28%)	238 (85.92%)	31 (67.39%) *	17.460 (3)	<0.001
Cellular internet access, n (%) Yes	62 (95.38%)	122 (91.04%)	182 (77.45%) *	17 (54.84%) *	34.621 (3)	<0.001
Cellular internet access, n (%) No	3 (4.62%)	12 (8.96%)	53 (22.55%) *	14 (45.16%) *		
Smartphone possession, n (%) Yes	65 (100.00)%	124 (93.23)% *	224 (95.73%)	24 (82.76%) *	12.212 (3)	0.004
Social media use frequency, Median (IQR) ^b^	4 (1)	4 (2)	3 (3) *	1 (3) *	H(3) = 51.359	<0.001

^a^ Unless otherwise noted; H indicates that Kruskal–Wallis test was used; NA = not appropriate for statistical analysis due to small cell sizes; ^b^ Range: Never (0) to Almost Every Day (4); IQR = interquartile range; * significantly differs from Millennials after Bonferroni correction (*p* < 0.016; *p* = 0.050/3), also denoted by shaded cell. n may not add up to total group size due to missing data.

**Table 5:** Digital health engagement by caregiver generation.

	Millennials	Gen X	Baby Boomers	Silent Gen	
	n = 73	n = 149	n = 277	n = 46	X^2^ (df) ^a^
Health/wellness app use, n (%) Yes	47 (67.14%)	99 (70.21%)	130 (50.78%) *	15 (42.86%) *	19.802 (3) **
Wearable health device use, n (%) Yes	40 (54.79%)	71 (47.65%)	85 (30.68%) *	6 (13.04%) *	27.839 (3) **
Social media peer health interaction, Median (IQR) ^b^	0 (1)	0 (1) *	0 (0) *	0 (0) *	H(3) = 27.562 **
Social media health-related videos, Median (IQR) ^b^	2 (2)	1 (2)	1 (1) *	0 (1) *	H(3) = 43.808 **

Data presented as n (% of total generation group, excluding participants with missing data for the item), unless otherwise noted; ^a^ unless otherwise noted; H indicates that Kruskal–Wallis test was used; ^b^ Range: Never (0) to Almost Every Day (4); * significantly differs from Millennials after Bonferroni correction (*p* < 0.016; *p* = 0.050/3), also denoted by shaded cell; ** denotes *p* < 0.001 for main omnibus test across groups; n may not add up to total group size due to missing data.

**Table 6:** Telehealth engagement in previous year by caregiver generation.

		Millennials	Gen X	Baby Boomers	Silent Gen	
		n = 73	n = 149	n = 277	n = 46	X^2^ (df)
**Telehealth Use Types: Used the internet to…**	Look for health or medical information, n (%)	60 (92.31%)	124 (91.18%)	225 (94.54%)	28 (90.32%)	1.943 (3)
	Send a message to a healthcare provider, n (%)	48 (73.85%)	103 (75.74%)	181 (76.05%)	18 (58.06%)	4.829 (3)
	View medical test results, n (%)	56 (86.15%)	105 (77.21%)	192 (81.01%)	19 (63.33%)	7.345 (3)
	Make an appointment with a healthcare provider, n (%)	53 (81.52%)	91 (66.91%)	154 (64.71%) *	14 (45.16%) *	13.263 (3) **
**Received Telehealth Care**	No, n (%)	32 (44.44%)	67 (45.58%)	137 (50.18%)	21 (46.67%)	1.259 (3)
	Yes, phone and/or video, n (%)	40 (55.56%)	80 (54.42%)	136 (49.82%)	24 (53.33%)	
**Offered Telehealth But Declined Because…**	In-person preference, n (%) Yes	10 (76.92%)	12 (92.31%)	33 (97.06)%	4 (100.00%)	NA
	Privacy concerns, n (%) Yes	4 (30.77%)	1 (7.69%)	4 (11.76%)	1 (15.63%)	
	Anticipated technology difficulty, n (%) Yes	2 (15.38%)	3 (23.08%)	7 (20.59%)	1 (25.00%)	
**Chose Telehealth Because…**	Provider recommended/required, n (%) Yes	29 (72.50%)	57 (70.37%)	92 (74.19%)	13 (61.90%)	1.458 (3)
	Wanted advice re: in-person care, n (%) Yes	13 (32.50%)	21 (26.25%)	30 (24.19%)	4 (20.00%)	1.466 (3)
	Avoid possible infection, n (%) Yes	20 (50.00%)	44 (54.32%)	64 (51.61%)	11 (52.38%)	0.241 (3)
	More convenient, n (%) Yes	27 (67.50%)	63 (77.78%)	72 (57.60%) *	9 (42.90%) *	13.190 (3) **
	Could include family or other caregivers, n (%) Yes	18 (46.15%)	19 (23.75%)	31 (24.00%)	7 (33.33%)	7.851 (3)

NA = not applicable due to small cell sizes; * significantly differs from Millennials after Bonferroni correction (*p* < 0.016; *p* = 0.050/3), also denoted by shaded cell; ** denotes *p* < 0.01 for main omnibus test across groups; n may not add up to total group size due to missing data.

## Data Availability

Data are available at https://hints.cancer.gov/data/default.aspx (accessed on 1 July 2023).

## Public Involvement Statement

Residents of the United States were randomly selected by address to participate. More detailed methodology is available from https://hints.cancer.gov/ (accessed on 1 July 2023).

## Guidelines and Standards Statement

This manuscript was drafted against the STROBE checklist for cross-sectional studies.
